# Decision Analysis of Disturbance Management in the Process of Medical Supplies Transportation after Natural Disasters

**DOI:** 10.3390/ijerph15081651

**Published:** 2018-08-03

**Authors:** Yuhe Shi, Zhenggang He

**Affiliations:** School of Transportation and Logistics, Southwest Jiaotong University, Chengdu 610031, China; SHI681242@163.com

**Keywords:** natural disasters, medical supplies transportation, cold-chain distribution, disturbance management, hybrid genetic algorithm

## Abstract

Public health emergencies, such as casualties and epidemic spread caused by natural disasters, have become important factors that seriously affect social development. Special medical supplies, such as blood and vaccines, are important public health medical resources, and the cold-chain distribution of medical supplies is in a highly unstable environment after a natural disaster that is easily affected by disturbance events. This paper innovatively studies the distribution optimization of medical supplies after natural disasters from the perspective of disturbance management. A disturbance management model for medical supplies distribution is established from two dimensions: time and cost. In addition, a hybrid genetic algorithm is introduced to solve the model. Disturbance recovery schemes with different weight coefficients are obtained through the actual numerical experiments, and experimental results show the effectiveness of the proposed model and algorithm. Finally, we discuss the formulation of weight coefficients in the case of emergency distribution and general distribution, which provide a reference for emergency decisions in disturbance events.

## 1. Introduction

In recent years, various natural disasters have occurred frequently, such as Hurricane Katrina in 2005, the Wenchuan Earthquake in 2008, and the Typhoon in the Philippines in 2013 [1,2,3,4]. After natural disasters, special medical supplies such as blood and vaccines are the key to reducing casualties and fighting infectious diseases. The efficient distribution of these special medical supplies is of great importance to public health and individual health. In general, special medical supplies, such as blood and vaccines, are extremely sensitive to temperature, and the quality of their cold-chain distribution is positively correlated with medical efficacy [5]. Only under specific temperatures or external environment conditions can it be ensured that medical supplies will not lose efficacy or deteriorate. In the circulation process of medical supplies, the cold-chain logistics are clearly important for ensuring the immune efficacy and safety of medical supplies [6]. However, the process of cold-chain distribution, which has the characteristics of high uncertainty, dynamics, and interactions, is easily affected by a multitude of disturbance events, including demand changes, road interruptions caused by natural disaster, vehicle refrigeration equipment failure, and so on, thus leading to the original distribution plan being affected, and even interruptions to the cold-chain.

Therefore, it is important to address disturbance events scientifically after the occurrence of natural disasters. After a disturbance event occurs, the distribution order of the remaining service objects should be adjusted, which is bound to result in a chain reaction and cause system confusion. At this point, we need to consider the impact of the disturbance on the entire cold-chain logistics and distribution system to generate an adjustment program that minimizes the system disturbance. Based on this, if the distribution quality and efficiency of medical supplies need to be ensured simultaneously, the medical supplies’ cold-chain distribution problem will become more complicated. How to effectively address disturbance events that lead to the interruption of the cold-chain and maintain the safety and efficiency of medical supplies are urgent problems that need to be solved in medical supply cold-chain distribution after natural disasters. 

The remaining parts of this paper are organized as follows. In the next section, a literature review on the disturbance management problem as well as the logistics and distribution of medical supplies is presented. Section 3 discusses the construction of the disturbance management model for medical supplies distribution (DMMSD). The hybrid genetic algorithm is introduced to solve the model in Section 4. Section 5 gives a numerical experiment and results analysis. Finally, Section 6 concludes this paper and presents expectations for future work.

## 2. Literature Review 

Since the main idea of the current research is to study the distribution optimization of medical supplies after natural disasters from the perspective of disturbance management, we review the studies in two fields: disturbance management in transportation and distribution optimization of medical supplies.

### 2.1. Disturbance Management in Transportation

There have been a large number of disturbance events across all walks of life. At present, disturbance management has become a hot issue for scholars to study, including aspects such as aviation disturbance management [7,8], supply chain disturbance management [9,10], machine scheduling disturbance management [11,12], railway scheduling disturbance management [13], and so on. As early as the 1970s and 1980s, research on disturbance had begun. The disturbance management was first applied in the aviation field by Yu [14], and since then, many research results have been produced on this classical optimization problem. There have also been many achievements in research on the disturbance management of logistics distribution. Zeimpekis et al. [15] classified the disturbance problem in logistics distribution and set up a mathematical model with the objectives of minimizing the delay cost and serving the largest number of customers. A disturbance recovery model of logistics distribution was established by Potvin et al. [16] to solve the problems of new customer demand and travel time disturbance, and they introduced an insertion algorithm for this model. Tiguiguchi and Shimamoto [17] studied the influence of uncertain vehicle traveling time on formulating a distribution plan and conducted an experiment that introduced changeable traveling time as the disturbance variable. Ruan and Wang [18] constructed a disturbance recovery model for the joint delivery of emergency medical supplies to analyze the disturbance of an emergency logistics system due to transfer point changes, and they designed a genetic algorithm to solve the model. Ding et al. [19] measured disturbance based on the prospect theory, and a multi-objective disturbance management model was proposed. Combined with related theories in behavioral science, Liu et al. [20] discussed the influence of disturbance events on an emergency logistics system from three aspects: demand point, decision maker, and logistics worker. In conclusion, many scholars have studied disturbance management in different transportation environments, but there is no literature related to disturbance events in the transportation of medical supplies.

### 2.2. Distribution Optimization of Medical Supplies

Another study related to this article is the logistics and distribution of medical supplies which requires high safety and punctuality. Based on the needs of theoretical research and practical application, scholars have conducted a great deal of research on this pertinent problem. According to the characteristics of emergency medical blood, Ramezanian et al. [21] proposed an optimization model for blood supply chain design in both deterministic and robust environments, and the application of the proposed model was evaluated by a case study in Tehran. The target functions in the integrated optimization model for the selection of emergency blood transfer points and transport routes proposed by Wang et al. [22] included having a minimum arrival time for emergency blood, maximum freshness at the time of reception, and minimization of the total transportation cost. A genetic algorithm for local neighborhood optimization was designed in their study. Chen et al. [23] considered the time constraints from the perspective of joint distribution and established an optimization model for multi-species cold-chain vaccine distribution. To minimize the maximum arrival time and the average arrival time, Campbell et al. [24] set up a path optimization model for vehicles with emergency supplies and used a local search algorithm to solve the model. Taking the Haiti earthquake in 2010 as an example, Battini et al. [25] developed a last mile distribution optimization model for emergency supplies and analyzed the optimization results under different scenarios. Ruan et al. [26] presented a two-stage approach for the intermodal transportation of medical supplies by “helicopters and vehicles” in large-scale disaster responses. Although there has been a large number of studies on the distribution optimization of medical supplies, few scholars have studied the distribution optimization of special medical supplies after natural disasters based on the perspective of disturbance management.

In short, as important medical supplies that are relevant to public health, the cold-chain distribution environments of blood and vaccine are highly unstable and vulnerable to disturbance. However, there have been few studies on the disturbance management of medical supply distribution. Taking vehicle breakdown (failure of refrigerating equipment) in the distribution process as an example, the idea of disturbance management is used to study the disturbance events in the cold-chain distribution of medical supplies in this paper. In contrast to the single-objective optimization model, we measure the disturbance from two dimensions, time and cost, and establish a disturbance management model for medical supply distribution (DMMSD) with minimum cost and time disturbance as the objective functions, and we design a hybrid genetic algorithm to solve this problem. Then, based on an actual case, the distribution schemes of disturbance recovery under different weights are obtained by our model and algorithm, thus providing a reference for the decision-making process of medical supply cold-chain distribution disturbance management.

## 3. Model Formulation

### 3.1. Problem Description

After a disaster, the rescue work in the first phase mainly involves the repair of basic facilities, such as road traffic and communication, as well as the simultaneous rescue of survivors. Then, on the basis of unimpeded communication and roads, a large number of medical materials are transported into the disaster area to reduce casualties and reduce the risk of secondary disasters caused by the outbreak of an epidemic situation [27,28]. This is called the second phase of post-disaster rescue [26,29], which is the background of this paper and the application environment for the DMMSD.

The problem studied in this paper can be described as follows. In the second phase of rescue after a natural disaster, the Medical Supplies Distribution Center (MSDC) distributes medical supplies to a number of temporary medical points (TMPs) through refrigerated trucks, and the locations of TMPs are known. There are same types of refrigerated vehicles, and medical supplies that require the same distribution temperature are transported by the same vehicle. Each refrigerated vehicle starts from the MSDC and will return to the MSDC after delivering the medical supplies to the designated TMPs along a known distribution route. The TMPs have time windows in which they receive services, which means the medical supplies are required to reach the TMP within a certain time interval. In the absence of any disturbances, the initial known delivery schemes can meet the requirements of the TMPs and the load limits of the vehicles. When a disturbance event occurs (taking vehicle breakdown and refrigeration equipment failure during the delivery process as an example), the problems that need to be resolved are as follows: the recovery of normal operation of the system as soon as possible, the completion of the distribution tasks with consideration of the interests of multiple stakeholders, and the minimization of the time disturbance and cost at the same time.

After a natural disaster, during the process of cold-chain distribution, any one of many factors, such as vehicles, cargoes, paths, demands and others, may be disturbed, which will influence the distribution task. Disturbance events can interrupt the cold-chain. If we continue to deliver medical supplies according to the initial schemes formulated before the occurrence of the disturbance event, inevitably some of the demand points will not receive the expected service, and the immune efficacy of the medical supplies will be affected. Therefore, it is necessary to construct a disturbance management model and to adjust the distribution schemes according to the disturbance event to utilize adjustment schemes that minimize the disturbance of the system. In this paper, disturbance management in the cold-chain logistics distribution of medical supplies is studied with the example of vehicle breakdown in the distribution process.

In short, the real environment after natural disasters is more complicated. In order to model the situation after a natural disaster and make scientific quantitative decision analysis, we refer to the literature [30,31,32,33,34,35,36,37,38,39,40,41,42], and make the following assumptions:

- As mentioned above, after a natural disaster, any one of a number of factors, such as vehicles, cargoes, paths, demands, and others, may be disturbed in the process of cold-chain distribution, which will influence the distribution task. In this paper, we assume that the transport capacity is disturbed (vehicle breakdown) to study the distribution optimization after a single type of disturbance event occurs.
- After a natural disaster, the rescuers dispatched by the government restore basic communication and traffic as soon as possible and rescue the survivors simultaneously. On the basis of unimpeded communication and roads, medical supply distribution can be carried out to ensure timely medical assistance to the injured and to reduce the risk of secondary disasters caused by the outbreak of epidemic situation. So, in this paper, we assume that the communication and roads are basically unimpeded during the distribution process of medical supplies.
- The location and service time windows of TMPs are known.
- The initial distribution scheme is known, and the same type of refrigerated vehicle is used.
- In a distribution task, each TMP is only served once.

### 3.2. Disturbance Processing Strategy

In the distribution process of medical supplies, a vehicle is not able to continue driving and the refrigerator cannot continue to work normally—the vehicle breaks down and the issue cannot be resolved in a short period of time. After the vehicle fails, the pending delivery cargoes and unfinished delivery tasks will be affected. The subsequent rescue mission includes picking up the cargoes and completing the unfinished delivery tasks of the disturbed vehicle. If a disturbance occurs to a distribution vehicle while the vehicle is on its way to the next TMP or the vehicle is servicing a TMP, it is assumed that the location of the disturbed vehicle is the pseudo demand point and the rescue vehicle needs to reach this location for rescue. If the vehicle fails when it is still at the MSDC, only new vehicles can be dispatched to service the affected demand points.

**Figure 1:**
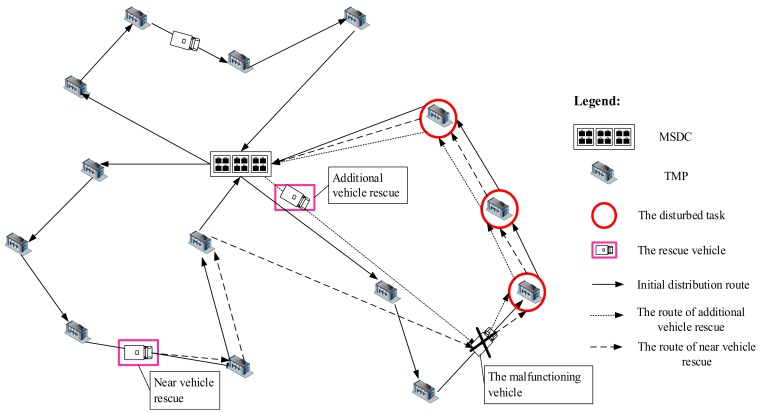
A schematic diagram of vehicle breakdown rescue.

The problem studied in this paper involves the disturbance management of the vehicle routing problem with time window (VRPTW); the cargoes need to be delivered in the time window required by TMPs. Furthermore, because of the characteristics of the medical supplies, it is necessary to store and transport them under certain temperature conditions. Thus, there are two kinds of rescue strategy: (1) the additional vehicle rescue strategy, which means new vehicles in the MSDC are dispatched for rescue in accordance with the original path, and (2) the near vehicle rescue strategy, which assumes that the locations of the vehicles on the way to deliver cargoes are pseudo distribution centers when the disturbance event occurs, and that the vehicles in the pseudo distribution centers are deployed to assist the disturbed vehicle to complete the remaining tasks. A schematic diagram of vehicle breakdown rescue is shown in Figure 1.

### 3.3. Parameters and Variables

According to the needs for building the model, this paper uses the following parameters and variables, as shown in Table 1.

### 3.4. Measurement of Disturbance

In this paper, the disturbance of vehicle breakdown is measured from two aspects: the arrival time disturbance of the medical supplies and the cost disturbance of the distribution. Then, a multi-objective function model with minimum cost and time disturbances is established.

#### 3.4.1. The Cost Disturbance 

After a disturbance event occurs, the original distribution plan is terminated, and a new distribution plan is started. The cost disturbance is composed of the cost of the path change, the cost of the additional new vehicle rescue, and the penalty cost that includes the penalty cost of failing to serve the TMPs and the cost of breaking the required time window. We set $C_{1}$ as the unit cost for a new vehicle; $C_{2}$ as the unit penalty cost for distribution failure; $\mu_{1}$ as the waiting cost per unit of time when the vehicle arrives at the TMP in advance; and $\mu_{2}$ as the penalty cost per unit of time when the vehicle is late to the TMP. The expression for cost disturbance is

(1) $$\begin{array}{cc} \min C & =\sum_{k\in R}\sum_{i\in RF}\sum_{j\in E_{0}}z_{ijk}C_{ij}d_{ij}L_{ijk}+\sum_{k\in D}\sum_{j\in RF}z_{0jk}C_{1}L_{0jk}+\sum_{k\in V}\sum_{i\in RF}\sum_{j\in E_{0}}C_{2}\left(1-x_{ijk}\right)\\ & +\sum_{k\in V}\sum_{i\in RF}\left(\mu_{1}\max\left\{ET_{i}-DT_{ki}{}^{\prime },0\right\}+\mu_{2}\max\left\{DT_{ki}{}^{\prime }-LT_{i},0\right\}\right) \end{array}\;$$

In Formula (1), $\max\left\{ET_{i}-DT_{ki}{}^{\prime },0\right\}$ indicates the advanced arrival time for vehicle k with service TMP i, and $\max\left\{DT_{ki}{}^{\prime }-LT_{i},0\right\}$ indicates the amount of time by which vehicle k is late to service TMP i.

#### 3.4.2. The Time Disturbance

After a natural disaster, there are three situations for the time disturbance of medical supplies arriving at the TMP: (1) the arrival time after adjusting the distribution scheme ($DT_{ki}{}^{\prime }$) is exactly the same as the original planned arrival time ($DT_{ki}$); (2) the arrival time after adjusting the distribution scheme ($DT_{ki}{}^{\prime }$) is later than the original planned arrival time ($DT_{ki}$); or (3) the arrival time after adjusting the distribution scheme ($DT_{ki}{}^{\prime }$) is earlier than the original planned arrival time ($DT_{ki}$). The third situation has a positive impact on the TMP, but it is likely generated by delaying the delivery time of other TMPs or increasing delivery vehicles, so we also regard it as a disturbance.

According to the analysis presented above, the disturbance of the medical supplies’ arrival time for TMP i can be expressed as

(2) $$\lambda \left(DT_{ki}-DT_{ki}{}^{\prime }\right),\;\lambda \in \left\{-1,0,1\right\}\;$$

where λ is a symbolic variable. When $DT_{ki}{}^{\prime }=DT_{ki}$, there is no arrival time disturbance in TMP i (λ=0); when $DT_{ki}{}^{\prime }>DT_{ki}$, that is, the arrival time after adjusting the distribution scheme is later than the originally planned arrival time (λ=1); when $DT_{ki}{}^{\prime }<DT_{ki}$, that is, the arrival time after adjusting the distribution scheme is earlier than the originally planned arrival time (λ=−1). Then, the arrival time disturbance of all of the TMPs can be obtained.

(3) $$\min T=\sum_{k\in RF}^{}\sum_{i=E^{ki}}\lambda \left(DT_{ki}-DT_{ki}{}^{\prime }\right),\;\lambda \in \left\{-1,0,1\right\}\;$$

where $E^{ki}$ is a collection of TMPs serviced by vehicle k in the original scheme.

### 3.5. The DMMSD Model Setting

In the second phase of post-disaster rescue, time and cost are the main targets followed by the three subjects of medical supplies distribution (MSDC, TMP, and distribution operator) in the face of a disturbance. Based on the analysis of disturbance in Section 3.4, the DMMSD model was constructed as follows:

(4) $$\begin{array}{cc} \min C & =\sum_{k\in R}\sum_{i\in RF}\sum_{j\in E_{0}}z_{ijk}C_{ij}d_{ij}L_{ijk}+\sum_{k\in D}\sum_{j\in RF}z_{0jk}C_{1}L_{0jk}+\sum_{k\in V}\sum_{i\in RF}\sum_{j\in E_{0}}C_{2}\left(1-x_{ijk}\right)\\ & +\sum_{k\in V}\sum_{i\in RF}\left(\mu_{1}\max\left\{ET_{i}-DT_{ki}{}^{\prime },0\right\}+\mu_{2}\max\left\{DT_{ki}{}^{\prime }-LT_{i},0\right\}\right) \end{array}\;$$

(5) $$\min T=\sum_{k\in RF}^{}\sum_{i=E^{ki}}\lambda \left(DT_{ki}-DT_{ki}{}^{\prime }\right),\lambda \in \left\{-1,0,1\right\}\;$$

subject to

(6) $$\sum_{i\in RF}\sum_{j\in E_{0}}x_{ijk}*g_{i}^{EP}\leq Q_{k}^{EP}\;\forall k\in V\;$$

(7) $$\sum_{j\in E_{0}}\sum_{k\in V}x_{ijk}\leq 1\;\forall i\in RF\;$$

(8) $$\sum_{k\in V}\sum_{j\in RF}z_{0jk}\leq \left|D\right|\;$$

(9) $$L_{ijk}=\left\{ \begin{array}{l} 1\;\left(i,j,k\right)\in L\left(EP\right)/L\left(OP\right)\\ 0\;\left(i,j,k\right)\in L\left(EP\right)\cap L\left(OP\right)\\ -1\;\left(i,j,k\right)\in L\left(OP\right)/L\left(EP\right) \end{array}\right.\;$$

(10) $$\sum_{i=1}^{a+b+1}x_{i\left(a+b+1\right)k}=1\;k=1,2,\ldots ,b\;$$

(11) $$\left(T_{0}-T_{n}\right)/t_{0}\geq DT_{ki}{}^{\prime }-t_{s},\;T_{n}\leq T_{0}\;$$

(12) $$\sum_{i=1}^{a+b+1}\sum_{k=1}^{b}x_{ijk}\left[t_{i}+w_{i}+\left(d_{ij}/s\right)\right]=t_{j}\;j=1,2,\ldots ,a+1\;$$

(13) $$ET_{i}\leq t_{i}+w_{i}\leq LT_{i}\;i=1,2,\ldots ,n\;$$

The model indicates that the objective of our problem is to minimize the deviation between the adjusted and initial schemes, which means that the degree of disturbance to the system is minimal, as shown in Expressions (4) and (5). Constraint (6) represents that the demands of TMPs cannot exceed the current load capacity of the rescue vehicle after the disturbance event occurs. Each task point can only be served once, and its operation is shown in constraint (7). The maximum number of available vehicles for rescue is emphasized in constraint (8). Constraint (9) imposes the notion of the definition of a path deviation parameter in the original and new schemes, including the path added in the new scheme, the path deleted in the original solution, and the changeless path. Constraint (10) shows the vehicle returning to the initial distribution center after finishing its service to the TMPs. Constraint (11) ensures that the disturbed vehicle is rescued before the medical supplies lose efficacy or deteriorate. The time window set for the TMPs must be met, which is imposed by constraints (12) and (13).

## 4. Algorithm Design

**Figure 2:**
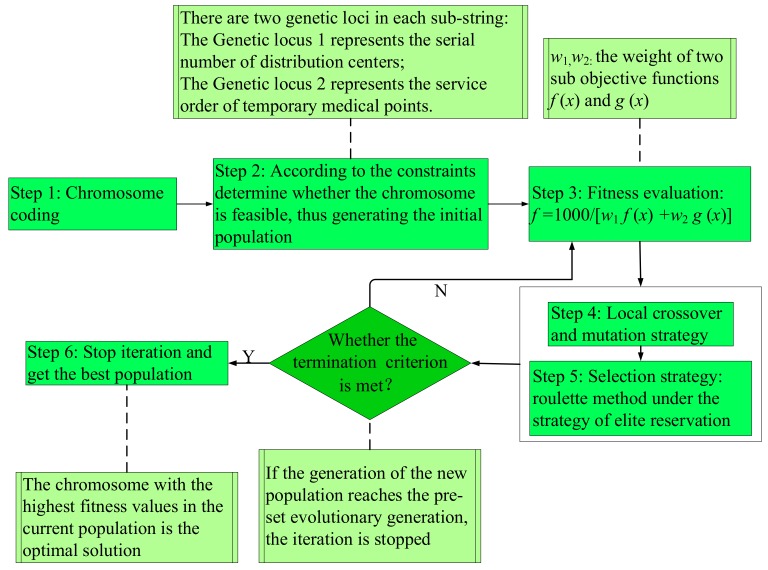
The specific process of the hybrid genetic algorithm.

Based on the idea of the genetic algorithm [43,44,45,46,47,48], a hybrid genetic algorithm (HGA) for solving the DMMSD model is designed in this section. The specific process is shown in Figure 2.

**Figure 3:**
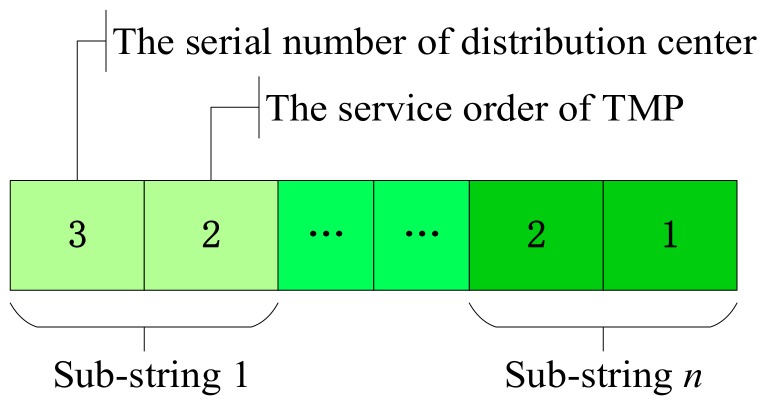
Example of chromosome coding.

Step 1: Chromosome coding. One chromosome represents a solution to the problem; each chromosome consists of n gene strings, and each gene string represents the service status of a TMP. There are two genetic loci in each substring: genetic locus 1 represents the serial number of the distribution center and genetic locus 2 represents the service order of TMP. Taking the chromosome in Figure 3 as an example, substring 1 indicates that TMP 1 is served by pseudo distribution center 3 in the second order, and substring *n* indicates that TMP *n* is served by pseudo distribution center 2 in the first order.

Step 2: Initializing the population. To ensure that the algorithm performs the optimization in a feasible solution space, each chromosome is decoded after it is generated in the process of population initialization. The chromosome is judged by constraints (6)–(13). If all of the constraints are satisfied, the chromosome is viable. Otherwise, new chromosomes will be regenerated through population initialization or genetic evolution until M chromosomes are obtained. 

Step 3: Evaluation of population fitness. In order to intuitively see the subtle changes in the fitness value in the algorithm convergence graphs, we set the numeric value of the numerator to 1000. The fitness function set in the HGA is as follows:

(14) $$f=\frac{1000}{w_{1}f(x)+w_{2}g(x)}\;$$

where $w_{1}$ and $w_{2}$ represents the weights of two sub-objectives, respectively.

Step 4: Crossover and mutation operation. According to the characteristics of the DMMSD model, we designed a local crossover and mutation method to improve the evolutionary efficiency of the algorithm. First, gene strings affected by disturbance are identified in two parent chromosomes. Then, the identified gene strings and unidentified gene strings are implemented in the local crossover (cross-probability, PC) and mutation operations (mutation probability, PM) separately to form progeny chromosomes. Finally, it is determined whether the newly generated progeny chromosomes meet the constraints until sufficient progeny chromosomes are constructed.

Step 5: Selection strategy. The roulette method under the strategy of elite reservation was chosen as the selection strategy in this paper. When generating the next-generation population, the parental population and the best individuals in the temporary population generated by crossover and mutation operations are directly reserved to the offspring. The other individuals in the new population are chosen from the parental and temporary populations by the roulette method.

Step 6: Termination conditions of the algorithm. The maximum iteration number of the genetic algorithm LS is set. The algorithm stops iterating when gen>LS, where gen is the algorithm iteration.

## 5. Numerical Experimental Design

The numerical experiments include the following three parts: First, the example data from the medical supplies distribution are used to verify the effectiveness of the DMMSD model in Section 5.1. Second, we obtain the experimental results and import them into the actual map to analyze the results in Section 5.2. Finally, the experimental results are discussed in Section 5.3.

This paper used MATLAB R2014a to implement the HGA, and all experiments in this paper were evaluated on PCs with Intel^®^ Core™ (Santa Clara, CA, USA) i7-3610QM CPU@ 2.10 GHz and 4 GB memory.

### 5.1. Model Experiment

#### 5.1.1. Experimental Parameters

We used a batch of medical supplies distribution data from a county MSDC that provides service for 20 township TMPs (due to the particularity of natural disasters, the acquisition of real data is difficult). Information about the MSDC and TMPs, such as the location, demand, and time window is shown in Table 2 (the MSDC is numbered 1 and the overall mass of the medical supplies is 47 kg per case). The parameters of the refrigerated vehicles are shown in Table 3 (the maximum load capacity, maximum travel speed, maximum load volume and other parameters of the vehicle will affect the final distribution plan), and the model parameters are set in Table 4. The average speed of the refrigerated vehicles was 30 km/h in the distribution process; 3 CNY/km was the transport cost for per unit mileage, and the maximum load of the refrigerated vehicle was 670 kg.

#### 5.1.2. Initial Distribution Scheme

**Figure 4:**
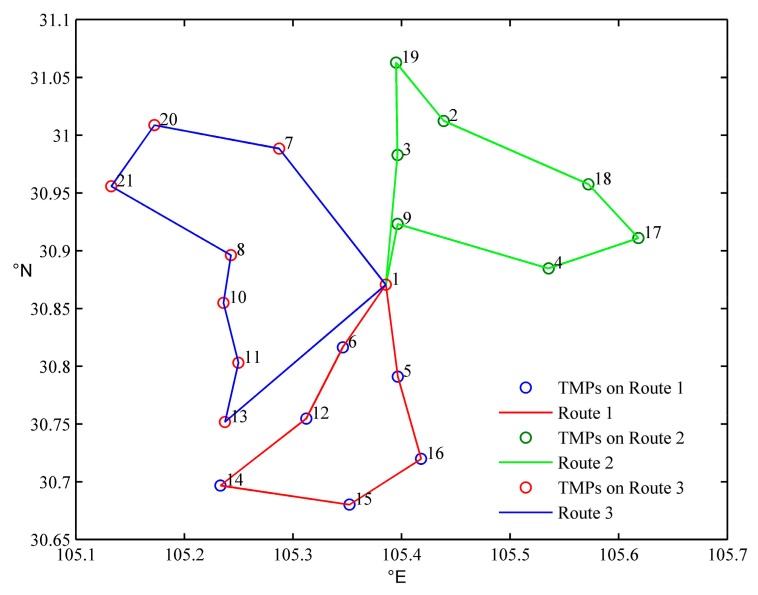
The initial distribution scheme.

The initial distribution scheme is shown in Table 5 and Figure 4.

#### 5.1.3. A Disturbance Occurs

**Figure 5:**
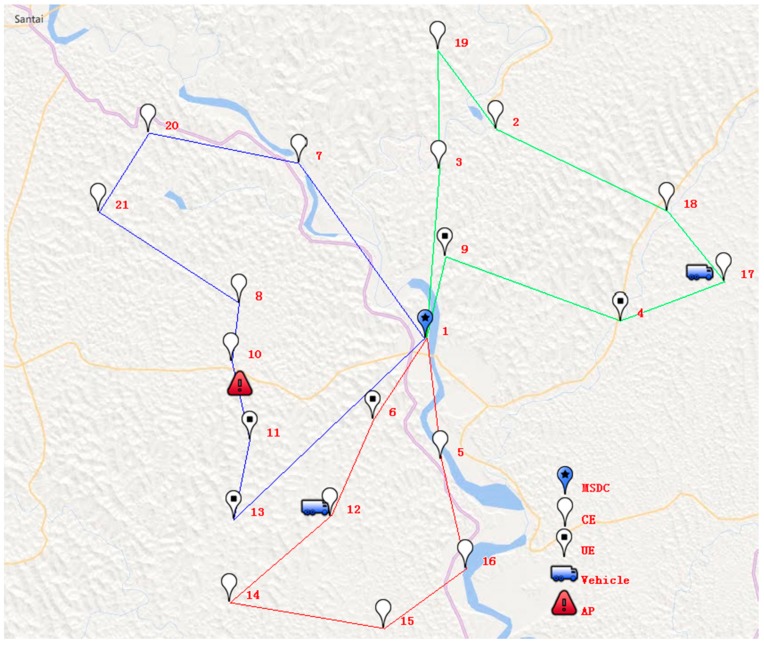
The state when the disturbance occurs.

The simulated situation was as follows: at 133 min after starting the delivery tasks, refrigerated vehicle 3 breaks down at position AP (105.242° E, 30.833° N), which is en route from TMP 10 to 11. CE represents the collection of TMPs that have been served when the disturbance event occurs; UE represents the collection of TMPs that have not been served when the disturbance event occurs; and VE represents the collection of pseudo demand points (AP∈VE). The state when the disturbance occurs is shown in Figure 5.

### 5.2. Experimental Results

#### 5.2.1. Results under Different Objective Weights

**Figure 6:**
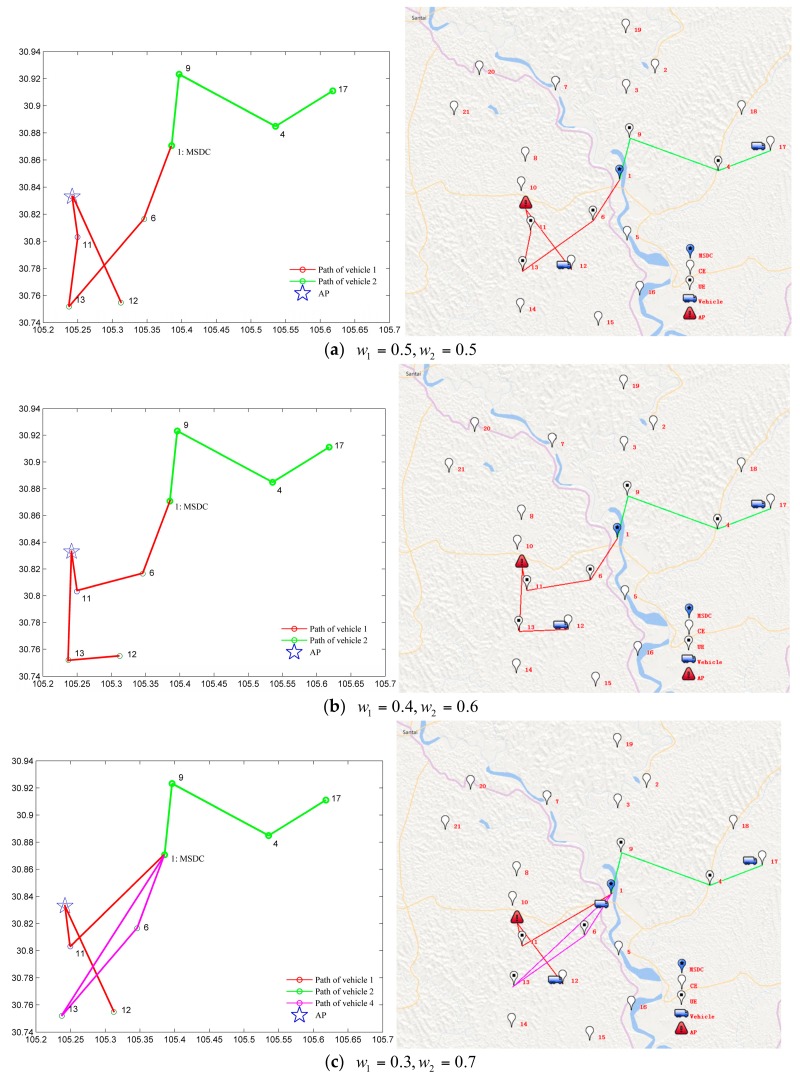

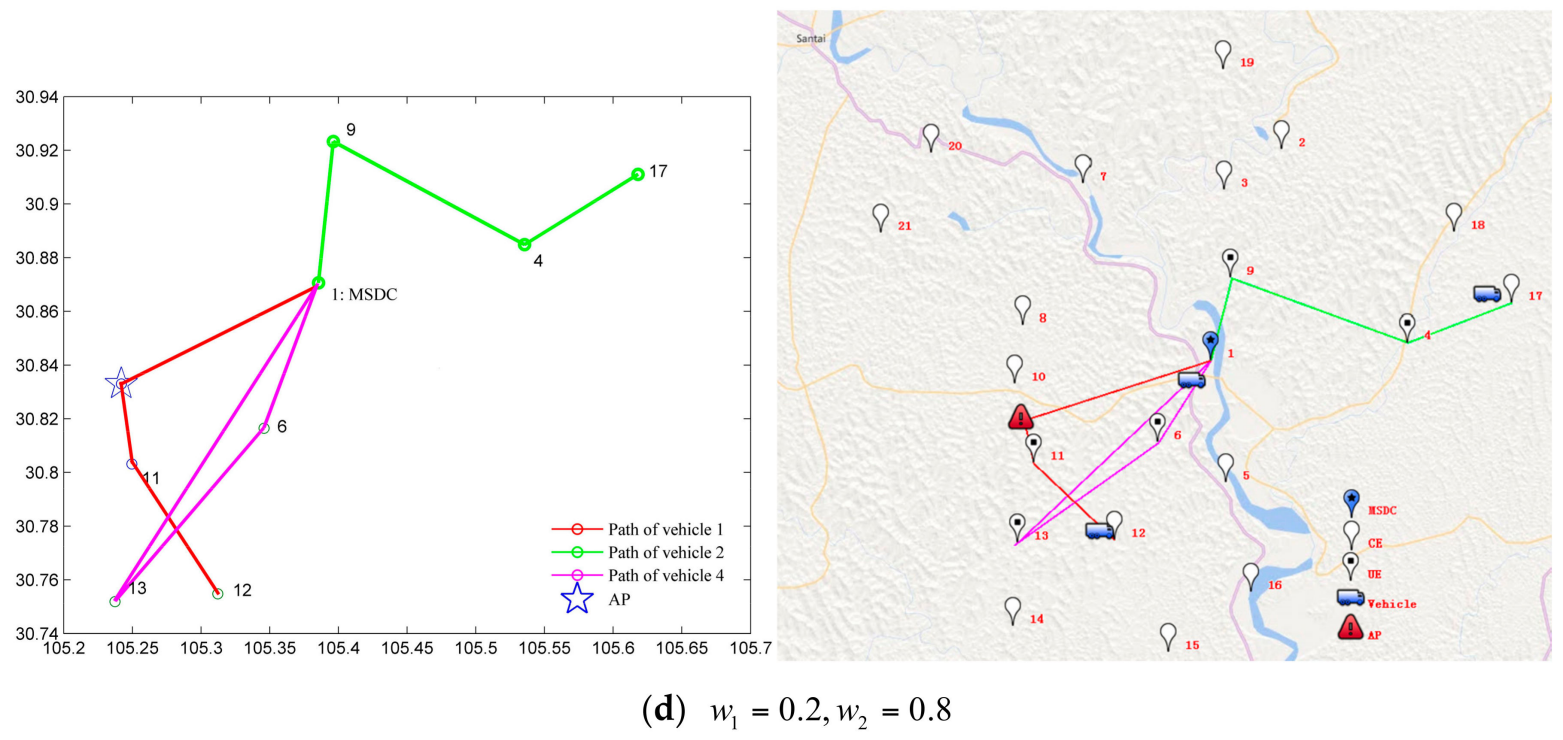
Distribution schemes under different objective weights.

After the disturbance event occurs, the DMMSD model proposed in this paper was used to obtain a rescue scheme for the TMPs that had not been served. The transfer time of the medical supplies was 10 min. The results under different weights of the objective functions were obtained, which are presented in Table 6 and Figure 6. Each group of data is iterated ten times, and the best solution is obtained as shown in Table 6.

From the results in Table 6 and Figure 6, we drew the following conclusions:

(1) Different weight coefficients of the objective functions correspond to different disturbance recovery schemes. As shown in Table 6, we set the weight coefficients of the time and cost objective functions to four different sets of numerical values ($w_{1}=0.5,\;0.4,\;0.3,\;0.2$; $w_{2}=0.5,\;0.6,\;0.7,\;0.8$); then, four different disturbance recovery distribution schemes were obtained.
(2) The number of vehicles used in different disturbance recovery distribution schemes is discrepant. As shown in Table 6, there were different numbers of vehicles in the four distribution schemes. The original distribution vehicles continued to be used in the first two distribution schemes; vehicle 1 was dispatched to rescue disturbed vehicle 3 and was responsible for the remaining TMPs that had not been served in the delivery tasks of vehicles 1 and 3 (i.e., the red route in Figure 6a,b). Vehicle 4 was added in the latter two distribution schemes to assist vehicle 1 in completing the service to the remaining TMPs that had not been served in the delivery tasks of vehicles 1 and 3 (i.e., the red route belongs to vehicle 1 and the purple route belongs to vehicle 4 in Figure 6c,d).
(3) For the time disturbance subobjective, the disturbance gradually decreased with an increase in its weight, but the disturbance of the distribution cost gradually increased. As seen from the results in Table 6, f(x) gradually increased with the decrease in $w_{1}$, and g(x) continuously decreased with the increase in $w_{2}$. In other words, as the weight coefficient of the cost objective function decreased, the cost of the disturbance recovery distribution scheme gradually increased, but the disturbance of the rescue scheme to time gradually decreased.
(4) The different weight coefficient settings of objective functions resulted in the same fitness value. From the results in Table 6, we found that the same value of f could be obtained from different values of $w_{1},\;w_{2},\;f(x),\;g(x)$ by calculations using Formula (14). This means that the different weight coefficient settings of the objective functions have no influence on the fitness value of the optimal solution, but different values of cost and time disturbance subobjective functions are formed; in addition, different disturbance recovery schemes are obtained at the same time. Therefore, in the face of actual disturbance events, emergency rescue decision makers should set the appropriate weight combination of objective function according to the current disturbance situation.

#### 5.2.2. The Convergence of the Algorithm

**Figure 7:**
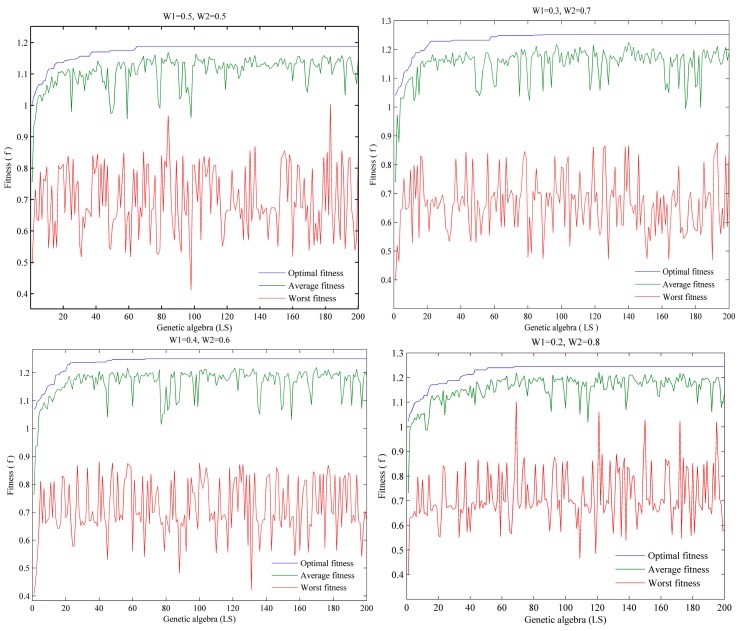
The convergence of the hybrid genetic algorithm (HGA) under different objective weights.

During the process of solving the results in Section 5.2.1, the convergence of the HGA under different objective weights is shown in Figure 7.

There are many factors that can affect the execution time of the algorithm, such as the size of the problem and the speed at which the computer executes the instructions (running memory, hardware quality, etc.). Therefore, when using different computer solutions, the solution time shown in Table 6 may be different. However, from Figure 7, we can clearly see that when solving the model with different weights, the HGA convergence effect was considerable, and the speed of convergence was fast. The optimal solution was obtained in the 60–80th generation, which proves that the algorithm has high stability.

### 5.3. Analysis of Experimental Results

Medical supplies are important public health supplies. Based on the concept of disturbance management, the DMMSD model was proposed to make emergency response decisions for disturbance events in the distribution process of these medical supplies. At the same time, we measured the disturbance from the two dimensions of time and cost, thus obtaining disturbance recovery route maps at the different weights to provide a reference for the decision-making of disturbance management scheduling in medical supplies distribution. However, during the actual distribution process, emergency decisionmakers responding to disturbance events need to make reasonable arrangements according to different emergency situations. 

After a disturbance event occurs, the rescue decision-makers can weigh the rescue time and cost according to the urgency and scope of the disaster situation. The research results of this paper can be used as a reference for decision makers' scientific decisions. The specific recommendations are shown below.

In an emergency distribution environment with a sudden epidemic situation or a large-scale natural disaster, public health supplies, such as vaccines and blood, need to rapidly be made available to meet the urgent demands of the TMPs. In this case, time is life. The MSDC needs to deliver the medical supplies to the TMPs in the shortest possible time to quickly control the epidemic situation or disease caused by natural disasters. Therefore, keeping the time disturbance of delivering medical supplies to TMPs at a minimum is the main goal. Thus, at this time, the time disturbance subobjective function has a very high weighting, and the cost is second.

However, the distribution of public health medical supplies, such as vaccines, can cause significant costs. Although all costs will be paid during the initial stage of an epidemic situation or large-scale natural disaster, the urgency for the demand of medical supplies will be gradually reduced after the epidemic situation is essentially stabilized or during the general distribution process. Meanwhile, there is a limitation of the actual delivery capacity; thus, what also needs to be considered is the operation costs of the logistics system. At this time, the weighting of the time disturbance is reduced and the weighting of the cost is increased.

## 6. Conclusions

Special medical supplies, such as blood and vaccines, are the key to reducing the number of casualties and controlling the epidemic after a natural disaster occurs. Therefore, it is obvious that the quality of distribution of medical supplies is important. In order to quickly respond to disturbance events during the distribution of medical supplies, a disturbance management model for medical supplies distribution was proposed in this paper to effectively deal with the interruption of cold-chain logistics caused by disturbance events and to ensure medical supply distribution is safe and effective. In this model, based on the concept of disturbance management, the measurement of disturbance is carried out from two dimensions: time and cost. The objective functions of the model are minimum cost and minimum time disturbance, and a hybrid genetic algorithm was designed to solve the model. Furthermore, disturbance recovery schemes under different weight coefficients were obtained through numerical experiments which provide a reference for the emergency decision makers of disturbance events to reasonably conduct the disturbance management. The validities of the model and algorithm were verified. 

In future research, we will consider further optimization of the distribution paths in the case of a combination of disturbances caused by different disturbance events. Meanwhile, real geographic situations should be taken into consideration in the problem of disturbance management.

## Figures and Tables

**Table 1:** The meanings of parameters and variables.

Parameters and Variables	Meaning
E	Collection of temporary medical points (TMPs)
CE	Collection of TMPs that have been served when a disturbance event occurs
UE	Collection of TMPs that have not been served when a disturbance event occurs
VE	Collection of pseudo demand points
V	Collection of distribution vehicles
R	Collection of vehicles on their way to deliver cargoes
D	Collection of vehicles at the Medical Supplies Distribution Center (MSDC)
0	Starting point of the vehicle
F	End point of the vehicle after the delivery service is completed
V=R∪D	Collection of all available vehicles
RF=UE∪VE	Collection of task points after the disturbance event occurs
$OP=\left\{\left(i,j,k\right)|\begin{array}{l} i,j\in E\cup 0\cup F,\\ k\in V \end{array}\right\}$	Original distribution scheme
EP	New distribution scheme, the collection of network nodes changes as $E_{0}=RF\cup \left\{0,F\right\}$
a	Total number of TMPs that have not been served
b	Total number of the vehicles on the way to deliver cargoes in the original distribution scheme
P	Collection of points, $P=\left\{p_{1},p_{2},\ldots ,p_{a+b+1}\right\}$, where $\left\{p_{1},p_{2},\ldots ,p_{a}\right\}$ represents the TMPs that have not been served; $p_{a+1}$ is the location of the disturbed vehicle; $\left\{p_{a+2},\ldots ,p_{a+b}\right\}$ represents the location of the vehicles on the way to deliver cargoes when a disturbance event occurs, that is, the pseudo distribution centers; $p_{0}$ is the location of the MSDC, which is the initial distribution center
$d_{ij}$	Distance between $p_{i}$ and $p_{j}$
s	Speed of the refrigerated vehicles
$t_{0}$	Time that the temperature inside the refrigerated box goes up by 1 °C when the refrigeration equipment fails to work
$T_{0}$	Critical temperature at which the medical supplies approach deterioration
$T_{n}$	Temperature inside the refrigerated box when the refrigerated vehicle is normal working
$w_{i}$	Service time of the vehicle for $p_{i}$
$t_{s}$	Moment of the disturbance event occurs
$DT_{ki}$	Time for vehicle k to reach TMP i in the original plan
$DT_{ki}{}^{\prime }$	Time for vehicle k to reach TMP i after adjusting the distribution scheme
$\left[ET_{i},LT_{i}\right]$	Time window of TMP i, including the beginning and end point of the arrival time required by the TMP
$g_{i}$	Medical supply demands of TMP i in the original distribution scheme
$g_{i}^{EP}$	Medical supply demands of TMP i after the disturbance event occurs
$Q_{k}$	Maximum load allowed for refrigerated vehicle k
$Q_{k}^{EP}$	Available load of vehicle k that is on its way to deliver cargo
$C_{ij}$	Transportation cost for a unit of distance for a refrigerated vehicle from TMP i to TMP j
L(EP)	Collection of distribution path edge in the new scheme
L(OP)	Collection of distribution path edge in the original scheme
$L_{ijk}$	The deviation parameter of the path, when (i,j,k)∈L(OP)/L(EP), $L_{ijk}=-1$ indicates the path edge between TMP i and TMP j is shown in the original scheme but not in the new scheme; when (i,j,k)∈L(EP)/L(OP),$L_{ijk}=1$ indicates the path edge between TMP i and TMP j is shown in the new scheme but not in the original scheme; when (i,j,k)∈L(EP)∩L(OP), $L_{ijk}=0$ indicates the path edge between TMP i and TMP j is shown in both the original and new schemes
$x_{ijk}$	A 0–1 variable, $x_{ijk}=1$ represents that vehicle k is driven from TMP i to TMP j, otherwise $x_{ijk}=0$
$z_{ijk}$	A 0–1 variable, $z_{ijk}=1$ represents that vehicle k is driven from the pseudo distribution center $p_{i}$ to demand point $p_{j}$, otherwise $z_{ijk}=0$
$z_{0jk}$	A 0–1 variable, $z_{0jk}=1$ represents that vehicle k is driven from the MSDC $p_{0}$ to demand point $p_{j}$, otherwise $z_{0jk}=0$

**Table 2:** Required information for MSDC and TMPs.

Number	Longitude (° E)	Latitude (° N)	Demand (Box)	Acceptable Time Window	Service Time (min)
1	105.385	30.871	0	5:30–17:00	0
2	105.439	31.012	1.5	6:00–8:00	10
3	105.396	30.983	0.5	7:30–9:00	5
4	105.535	30.885	1.5	6:00–8:00	10
5	105.396	30.791	1.5	6:30–8:20	10
6	105.346	30.816	1	7:40–9:30	8
7	105.287	30.989	1	7:00–9:00	8
8	105.243	30.896	0.5	7:20–9:00	5
9	105.396	30.923	1	7:30–9:00	8
10	105.236	30.855	0.5	7:00–8:30	5
11	105.250	30.803	1	7:30–9:30	8
12	105.312	30.755	2	7:30–9:30	15
13	105.237	30.752	0.5	7:30–9:30	5
14	105.233	30.697	1.5	7:30–9:30	10
15	105.352	30.680	1.5	7:30–9:00	10
16	105.418	30.720	1.5	6:50–8:30	10
17	105.618	30.911	1.5	7:00–8:40	10
18	105.572	30.958	1.5	7:00–8:40	10
19	105.395	31.063	0.5	7:50–9:00	5
20	105.172	31.009	1	6:30–8:30	8
21	105.133	30.956	1	7:50–9:00	8

**Table 3:** Vehicle Parameters.

Parameter	Parameter Value	Parameter	Parameter Value
Outline dimension	4845 × 2000 × 2500 (mm)	Container volume	3 m^3^
Fastest speed	135 km/h	Rated load capacity	670 kg
Engine type	SOFIM8140.43S4	Fuel type	diesel oil
Engine power	95 kw	Engine emission volume	2798 mL

**Table 4:** Model parameter settings.

Parameter	Parameter Value
$t_{0}$	15 min
$T_{0}$	8 °C
$T_{n}$	2 °C
$t_{s}$	133 min
$C_{1}$	300 CNY
$C_{2}$	1000 CNY
$\mu_{1}$	60 CNY/h
$\mu_{2}$	80 CNY/h
PC	0.8
PM	0.2
LS	200

**Table 5:** The initial distribution service order.

Route Number	Service Order
1	1-5-16-15-14-12-6-1
2	1-3-19-2-18-17-4-9-1
3	1-7-20-21-8-10-11-13-1

**Table 6:** Results under different objective weights.

Weights	$w_{1}=0.5,w_{2}=0.5\;$	$w_{1}=0.4,w_{2}=0.6\;$	$w_{1}=0.3,w_{2}=0.7\;$	$w_{1}=0.2,w_{2}=0.8\;$
The number of vehicles that complete the remaining distribution Tasks	2	2	3	3
Path of Vehicle 1	12-AP-11-13-6-1	12-13-AP-11-6-1	12-AP-11-1	12-11-AP-1
Path of Vehicle 2	17-4-9-1	17-4-9-1	17-4-9-1	17-4-9-1
Path of Vehicle 3	AP	AP	AP	AP
Path of Vehicle 4	/	/	1-13-6-1	1-13-6-1
f(x)/CNY	1526.1	1819.7	2400.4	3627.5
g(x)/min	153.7	103.4	99.7	80.5
Average time for solving (s.)	8.4	8.1	8.9	10.2
